# Therapeutic Effects of Pre-Gelatinized Maca (Lepidium Peruvianum Chacon) used as a Non-Hormonal Alternative to HRT in Perimenopausal Women - Clinical Pilot Study

**Published:** 2006-06

**Authors:** H. O. Meissner, H. Reich-Bilinska, A. Mscisz, B. Kedzia

**Affiliations:** 1 *Faculty of Health Studies, Charles Sturt University & Therapeutic Research, TTD International Pty Ltd, Sydney, Australia;*; 2 *Specialist Gynecology Clinic, Glogow, Poland;*; 3 *Research Institute of Medicinal Plants, 27 Libelta St., Poznan, Poland*

**Keywords:** alternative to HRT, blood hormones, Maca (Lepidium peruvianum), perimenopause

## Abstract

**Background:**

Roots of cruciferous plant Lepidium peruvianum Chacon cultivated in high plateaus of Andes and known under its common name Maca, have been traditionally-used as an energizing vegetable with therapeutic properties for both men and women. Maca has been recognized by natives of Peru as herbal remedy helping to treat conditions affecting menopausal women.

**Objective:**

The effects of Pre-Gelatinized Organic Maca (Maca-GO) on quantitative physiological responses and alleviation of symptoms contributing to menopausal discomfort in perimenopausal women was examined.

**Methods:**

In this, four months, double blind, crossover, randomized pilot trial, monthly measurements of the following blood serum constituents were taken: Estrogen (E2), Follicle Stimulating Hormone (FSH), Luteinizing Hormone (LH) and Progesterone (PGS), Cortisol (CT), Adrenocorticotropic Hormone (ACTH), Thyroid Hormones (TSH, T3, T4), minerals (Ca, K, Fe) and lipid profile (Triglicerides, Total Cholesterol, LDL, HDL). In monthly interviews conducted by gynecologist, body weight and blood pressure were registered and Menopausal Index according to Kupperman’s was determined. Toxicity of Maca -GO determined on rats showed its safe use at the level of 7.5mg/kg body weight. A group of 20 women (aged 41-50 years), who fulfilled criteria of being in perimenopausal stage (E2 above 40pg/ml and FSH below 30IU/ml), were randomly allocated to two even groups, one receiving for two months Maca-GO and the other Placebo capsules followed by a crossover with treatment change for another two months period. All participants signed informed consent to participate. Two 500mg hard capsules with Maca-GO or Placebo were self-administered by participants twice daily with meals (total 2g/day).

**Results:**

Two months administration of Maca-GO significantly alleviated symptoms of discomfort observed in majority of women involved in the study (74%-87%) as assessed by Kupperman’s Menopausal index. This was associated with significant increase in E2 and FSH, Progesterone and ACTH levels, and reduction in blood pressure, body weight, Triglycerides and Cholesterol levels. There was a distinctive placebo effect observed at the beginning of the study.

**Conclusions:**

The results showed that in addition to reduction in body weight, blood pressure and increasing serum HDL and Iron, pre-gelatinized Maca-GO may be a valuable non-hormonal plant preparation for balancing levels of hormones (FSH, E2, PG and ACTH) and alleviating negative physiological and psychological symptoms (frequency of hot flushes, incidence in night sweating, interrupted sleep pattern, nervousness, depression and heart palpitations) experienced by women in perimenopausal stage. It appears that Maca-GO may act as a toner of hormonal processes, leading to alleviation of discomfort felt by perimenopausal women, hence, its potential use as non-hormonal alternative to HRT program.

## INTRODUCTION

Roots of cruciferous plant Lepidium peruvianum Chacon were cultivated in high plateaus of Andes (above 4,200 m a.s.l.) since the times of Incas and are known until the present time by natives of Peru and internationally, under the common name of Maca. It has been traditionally used as an energizing vegetable with therapeutic properties of value for both men and women (1, 2). Amongst wide spectrum of traditional uses of Maca such as for: energy, hormone balancing, healthy thyroid functioning, sexual functioning, PMS, menopause, as well as is recommended for helping to maintain healthy bones, as a tonic for elderly and assisting in convalescence (1, 2). Maca has also been used by natives as herbal remedy which helps to treat conditions affecting menopausal women, particularly of value in treating depressive and stress-related symptoms - commonly experienced by women in menopausal stage.

According to Chacon (1), Maca - a native Peruvian plant, in addition to its high nutritive value has also number of traditionally-acknowledged therapeutic and medicinal applications, and after her pioneering dissertation (3) in which she has “re-discovered” Maca as a dietary supplement in its present form. Chacon (3) linked alkaloids as active Maca components to physiological regulating and stimulating action on ovaries and testes of rats. Now, Maca has attracted attention of researchers internationally, who try to link multiple therapeutic properties of this unique plant to its individual biochemical constituents. Over the last decade, number of publications provided botanical and genetic characteristics of Maca and reported on various biochemical and therapeutic properties of the root (4-10), which by now, is widely marketed as a dietary supplement in Peru and distributed by numerous trading and nutriceutic companies in the USA, Europe and Asia under its generic name of “Maca” and/or under various commercial trade mark names.

Maca, which unlike soy/genistein and black cohosh, contains no plant hormones (1, 4), for centuries has been successfully used by native people of Peru for hormonal imbalances, menstrual irregularities, fertility, and menopausal symptoms, including hot flashes, vaginal dryness, loss of energy, libido and depression (6). Since an introduction of Maca as a dietary supplement with therapeutic properties at the Anti-Aging Medical Conference held in the USA back in 1997 (5), it has been increasingly used as a medicinal herb by doctors practicing CAM (Complementary/Alternative Medicine) including its use as an alternative to and/or in reducing dependence of women on HRT program (6).

According to Chacon (1) and Muller (5), Maca works in an entirely different way for most women than phytoestrogen herbs (like black cohosh and licorice root) or HRT, by promoting optimal functioning of the hypothalamus and the pituitary, thereby improving the functioning of all the endocrine glands. Original research conducted by Chacon (3) reviled that, alkaloids in the Maca root, produced fertility effects on the ovaries and testes of rats. She has further deducted that the alkaloids were acting on the hypothalamus-pituitary axis, which may explain why the effects in humans are not limited to ovaries and testes, but also acts on the adrenals, giving a feeling of greater energy and vitality and on the pancreas and thyroid as well.

In previous papers from this series (11, 12), results of study on pre-gelatinized form of Maca root preparation (Gelatinized Organic Maca – Maca-GO), showed its positive physiological effect in both, clinical study on postmenopausal women (11) and in short- and long-term laboratory animals models with the use of bioassays on adult female and male rats (12). The observed effects on animals were gender and dose dependent, with different responses observed during a short- and a long-term Maca-GO administration (12). Extrapolating results of model laboratory assays to humans, those study demonstrated that Maca-GO may help women to reduce discomfort experienced not only during the menopause, but also, well before the onset and then at the time of entering and during menopausal stage. In addition to balancing effects of Maca-GO on hormones FSH, E2 and progesterone appropriate to the gender, in laboratory animals, positive results were also recorded in terms of tendency to restrict weight increase, lowering triglycerides in blood plasma and an increase in calcium and phosphorus deposition in bone and muscle tissues. It was reasonable to suppose that Maca-GO may be of value in alleviating perimenopausal symptoms as a potential substitute to HRT, or reducing dependence on HRT programs. Laboratory assays on male and female rats demonstrated also that Maca-GO may also be considered as a non-hormonal energizing supplement, assisting not only physically-active people and sportsmen, but in those women complaining on lack of energy and stamina, often experienced by women entering menopausal stage of life.

Since the first report of Chacon on medicinal properties of Maca root (3), it has been generally accepted and confirmed in number of studies, that this plant doesn’t contain plant estrogens or any other phytohormones (13-17), but through plant sterols, stimulate endocrine system helping to maintain hormonal balance (1) in a way that is not yet well understood (2, 18). According to Muller (6), these sterols are used by the body with the help of the pituitary to improve adrenal function, ovarian and testicular function, as well as the functioning of the thyroid and the pancreas, and the pineal gland. Multi-functional effect of Maca on endocrine relationships may also explain reported in the literature, its positive influence on stimulation of endocrine glands in regulation of hormonal balances in the body (4, 10) and particularly in women entering a perimenopausal state of life.

A pilot study conducted on early postmenopausal women (11), confirmed that through balancing hormones in the body, Maca-GO helped women to reduce discomfort which they experienced in early postmenopausal stage. Therefore, in this pilot clinical study, an attempt has been made to observe the effect of Gelatinized Organic Maca on changes in levels of gonadal, pituitary, thyroid and adrenal hormones as well as other biochemical blood indices in alleviating symptoms of discomfort as assessed by Menopausal Index in two randomly selected groups of perimenopausal women volunteers.

## MATERIALS AND METHODS

### Aim

The aim of this double blind, crossover, and randomized pilot clinical study was to determine the effect of the oral administration of capsules containing pre-gelatinized dried and pulverized hypocotyls of Maca (Lepidium peruvianum Chacon) on quantitative physiological responses and alleviation of symptoms contributing to menopausal discomfort in perimenopausal women.

### Maca

The plant species is described in details in monographs by Chacon (1) and Obregon (2) as well as in the catalogue of the plants and gymnosperms of Peru (19). Maca roots, used in the present study were cultivated, harvested and dried in Junin area (Central Andean Region of Peru between 4200m and 4500m altitude) and represented typical distribution of three main ecotypes (out of 13 known): black, yellow and purple/red roots observed in this cultivation area – averaging to approximately 16%, 48% and 9% respectively. After some three month of air drying at high altitude (on the plantation site), according to traditionally used system of roots dehydration, considered superior to oven dried method currently used in commercial “modern” operations in Peru (Obregon, 2001, 2006 and Chacon 2003, 2006 – personal communications), dried Maca roots selected for this study, were transported to a processing plant at the National Institute of Agricultural Research (NIAR), National Agricultural University La Molina in Lima (Peru) after previous attestation of its organic status (formal certification by SKAL as “organic”), and its authentication by Dr Gloria Chacon as cultivated Maca - Lepidium peruvianum Chacon, which represent the same plant species, which she used in her pioneering work on Maca published back in 1961 (3) and she used during more than 40 years of her research on this plant (1). Dry roots were transported to a processing plant at the National Institute of Agricultural Research, National Agricultural University La Molina in Lima, Peru (NIAR), where it was subjected to a gelatinization process followed by drying and pulverizing.

After cleaning (washing under pressure) and cutting into pieces, dried hypocotyls of Maca were re-hydrated prior to being exposed to a gelatinization process comprising of exposure to a short-term elevated pressure under moist conditions (a proprietary extrusion process), followed by drying and pulverizing. Such treatment of Maca, without any chemicals used in the process, resulted in the final powdered product (Maca-GO) achieving increased density and through pre-gelatinization of a starch component in the product (not less than 98% according to BRI Laboratory assay, Sydney, Australia), expected to promote its easier digestion and bio-availability. The processing of Maca was identical to the one described in the previous papers from this series (11, 12).

Composition of the pre-gelatinized Maca-GO powder (batch TTD-ZMP-20100351) as received directly from the manufacturing line in Peru (NIAR) was analyzed first by the NIAR Laboratory in Lima, and then the biochemical composition was verified by the Analytical Laboratory of the Research Institute of Medicinal Plants, Poznan, Poland (Table 1), prior to encapsulation and coding for use in the study. Maca-GO or Placebo powder (sorbitol & cellulose) were encapsulated in 500 mg quantity in identically-looking hard vegetable gelatin capsules.

**Table 1:** Composition of Pre-Gelatinized Maca-GO (*Lepidium peruvianum* Chacon)

No.	Specification	Unit per 100g of product	Pre-Gelatinized Maca Root Powder (Maca-GO)

1	Energy value	kJ kcal	1.235
2	Moisture	g	5.8
3	Ash	g	4.9
4	Crude Protein	g	11.7
5	Ether Extract	g	4.1
6	Carbohydrates Total	g	73.5
7	Available Carbohydrates	g	52.0
8	Dietary Fiber	g	21.5
9	Vitamin C	mg	659.3
10	Thiamine	mg	167.1
11	Calcium	mg	318
12	Phosphorus	mg	352
13	Sodium	mg	52
14	Potassium	mg	1373
15	Glucosinolates as Synigrine	mg	200
16	Unsaponified fraction	% oil fraction	16
17	Campestral	% unsuponified	7.8
18	Sigmasterol	% unsuponified	4.1
19	β-sitosterol	% unsuponified	24.2
20	Arginine	mg	300
21	Gelatinization Index^a^	%	98.5

a Degree of gelatinization of starch obtained as a result of extrusion process. Assay conducted using the method by the BRI Laboratory, Sydney, Australia.

### Toxicity (LD50)

Prior to the clinical study on perimenopausal women, toxicity of the batch of Maca-GO received for use in the study was determined on male and female rats according to Litchfield-Walloon’s method modified according to the harmonized OECD procedure (20). Experiment was conducted under a standard laboratory model approved by a Bioethics Committee for Animal Experimentation of the Research Institute of Medicinal Plants (RIMP) in Poznan.

### Subjects

Twenty Caucasian volunteers (women aged 41-50 years) were selected to this pilot clinical study. They represented healthy, regularly menstruating women (with history of no more than two periods missed during the 12 months prior to the study), who were not used or been involved in any hormonal treatment prior to the inclusion in this study and were not taking any medications for at least 6 months prior the study. A signed informed consent was obtained from all subjects regarding their voluntary participation in the trial conducted under specialist Gynecologist’s supervision in a Private Clinic in Glogow (Poland). All enrolled subjects were informed of the purpose, benefit and possible risks of the study.

### Experimental protocol

Hard gel capsules both placebo and Maca-GO (500 mg net per capsule), used in this study were custom-manufactured at the Research Institute of Medicinal Plants in Poznan. The capsules were kept in numbered containers.

The trial was of a double-blind, placebo-controlled, crossover design and randomization of treatment allocation was performed in blocks of two with the block details unknown to the investigators. The design has two immediate successive periods of two months duration each, with randomized allocation of the two Treatments (A and B) at the start of the study into Placebo group = PP or Maca-GO group = MM. Each Treatment has started with two initial months “before crossover” - without prior run-in period, followed by crossover (X) and the second two months “after crossover” - with a prior run-in period on either Maca-GO or Placebo. One of the responsible investigators enrolled all patients. The patients as well as research team were kept blind throughout the study.

During monthly interview with a doctor gynecologist who was one of the investigators, all subjects received a numbered container with a monthly allocation of capsules to be taken as a dose 4 × 500mg capsules (2,000 mg) daily, either Maca-GO, or Placebo. Capsules were self-administered according to the following schedule: 2 capsules some 30 minutes before the morning and 2 capsules before the evening meal for the period of four months – according to experimental cross-over design (Treatment A as a sequence PP-MM and B as a sequence MM-PP accordingly). The 2 g/day dose of Maca-GO was identical to the one applied in a previous study (11), and was adopted from practical clinical experience by Muller (5 and 6) who recommends such a dose for alleviation of menopausal discomfort to women in the USA.

With the start and then on the completion of each monthly interval of the study, all woman were interviewed by the gynecologist and requested to answer a set of standard questions to determine a Menopausal Index according to Kupperman’s questionnaire. At the same time, body weight and blood pressure were checked and blood was sampled for hormones and other biochemical analyses.

The study was carried out by specialist Gynecologist and researchers of the Research Institute of Medicinal Plants in Poznan under international supervision between January 2004 and June 2005.

### Assays

Blood serum level of hormones was measured on monthly basis: Estrogen (E2), Follicle Stimulating Hormone (FSH), Luteinizing Hormone (LH) and Progesterone (PGS) as well as, Cortisol (CT), Adrenocorticotropic Hormone (ACTH), Thyroid Hormones (TSH, T3, T4). Blood pressure, body weight, serum mineral contents (Ca, K, Fe) and lipid profiles were also determined in monthly intervals together with indices of menopausal discomfort determined in personal interviews by doctor with the use of questionnaire of Menopausal Index according to Kupperman.

Hormone assays were conducted by a Clinical Diagnostic Laboratory LABMED in Poland using officially accepted standard clinical procedure on Immulite – DPC equipment. Precision of this technique is monitored by National Center of Quality of Diagnostic Medical Laboratories in Poland and the Laboratory is a participant of the International Quality Control RIQAS maintained by Randox Company.

### Statistical analysis

Data were expressed in means and Standard Error of Mean (± SEM) used in statistical analysis performed by the use of Wilcoxon signed rank test. The difference were considered significant at *P*<0.05 and high significant at *P*<0.01 level.

## RESULTS

### TOXICITY (LD50) for Maca-GO

All animals survived the LD50 toxicity test without any adverse effects noticed on the basis of abnormal behavior and histopathology of internal organs (liver, spleen, pancreas and testis or ovaries). On the basis of the obtained results the LD50 for Maca-GO was >7.5 g/kg body weight (the highest dose applied in this study).

### Subjects’ participation

The study was started with 20 subjects out of which during the 4 months trial, 1 subject have failed to conclude the first two months of trial on Placebo (PP-MM) due to not being satisfied with the program and deciding to choose other therapy. The second person resigned from participation in the study after crossover in MM-PP group, prior to concluding the first month on Placebo treatment by failing to present herself for the monthly blood sampling and interview by the doctor. Data from these two women were therefore excluded from the final summary of results.

### Period “before crossover” (without prior run-in period)

**Figure 1:**
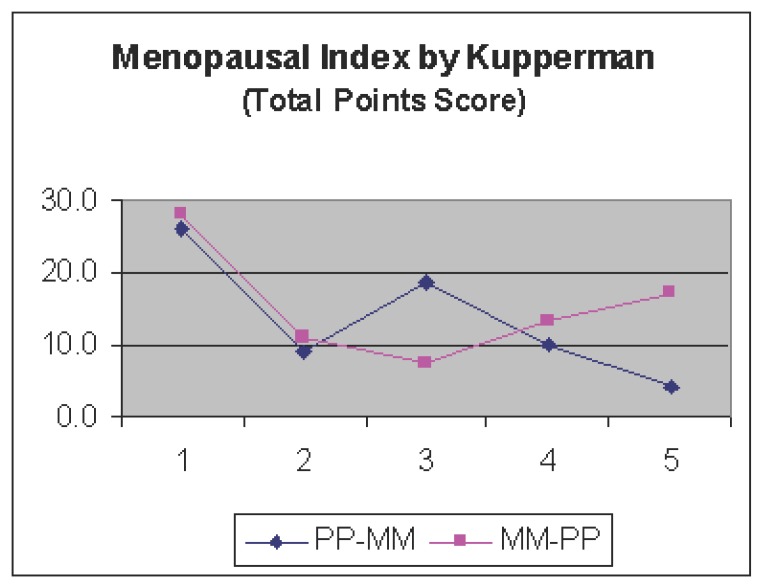
FSH, LH, E2 and PG levels in perimenopausal women (n=18): One group (PP-MM sequence) receiving for two months daily dose of 2 g (two 500 mg capsules twice daily) of Placebo (PP), followed by crossover (X) after which identical daily dose of Maca-GO (MM) was administered for another two months period (Treatment A - without prior run-in period), and the second group (MM-PP sequence) treated with identical daily doses of Maca-GO for two months followed by crossover and two months of Placebo administration (Treatment B - with prior run-in period)1. Blood sampling points: 1) Start - before the trial; 2) after subsequent one and 3) two months administration of PP or MM (Treatment A or B respectively), with crossover at the point 3 (X), followed by another two months (4 & 5) of administration of the same daily dose of MM or PP. Statistical significance has been determined according to multiple Wilcoxon’s test. * *P*<0.05; **P<0.01; NS, Not Significant.

Results from hormonal assays summarized in Figure 1 demonstrate that after one month of treatment, FSH and E2 in Placebo group noticeably increased (*P*>0.05) in similar manner as in Maca-GO group. Two months treatment resulted in FSH in Placebo group returning to the level at start of the trial while in Maca-GO group the FSH and E2 concentrations continue to increase. After two months of the Period A, the level of FSH was significantly higher (*P*<0.01) as compared to both starting point and the Placebo treatment, while similar changes in E2 were not statistically different (*P*>0.05).

During “before crossover” stage, Maca-GO treatment resulted in a substantial, but statistically not significant increase (*P*>0.05) in Progesterone and LH levels while Placebo group noticeably (*P*>0.05) lowered these measurements after the first month, returning after the second month of treatment, to the level close to the start of the trial. The differences between responses of perimenopausal women to Placebo and Maca-GO after the first and the second month in Period A were significant at *P*<0.05.level.

No significant differences were observed between the two groups in levels of LH along the “before crossover” stage, although, Maca-GO resulted in slight (*P*>0.05) increase after first and second month of treatment as compared to both, the starting point and Placebo treatment.

From results of other hormones summarized in Table 2, it appears that there were no statistical differences (*P*>0.05) observed in concentrations of the thyroid (TSH, T3 and T4) and adrenal hormones (Cortisol and ACTH), Maca-GO resulted in slight increase (*P*>0.05) in concentrations determined after the first and the second month of treatment, while Placebo had no visible effect.

**Table 2:** Levels of Thyroid and Adrenal Hormones in two groups of perimenopausal women (n=18): One group (PP-MM sequence) receiving for two months daily dose of 2 g (two 500 mg capsules twice daily) of Placebo (PP), followed by crossover (X) after which identical daily dose of Maca-GO (MM) was administered for another two months period (Treatment A - without prior run-in period), and the second group (MM-PP sequence) treated with identical daily doses of Maca-GO for two months followed by crossover and two months of Placebo administration (Treatment B - with prior run-in period)^a^

Hormone	Period on Maca-GO or Placebo (before - and after crossover)	Start of the Trial	without prior run-in period		X	with prior run-in period		SE (±) of Mean (significance)
			After 1 month	After 2 month	↑	After 3 month	After 4 month	

TSH	PP-MM	1.9	2.1	2.0	X	1.8	2.3	0.43
(mcIU/ml)	MM-PP	1.7	1.8	1.9	X	1.7	1.9	NS
T4	PP-MM	1.1	1.2	1.1	X	1.1	1.2	0.12
(ng/100ml)	MM-PP	1.0	1.0	1.0	X	1.0	1.1	NS
T3	PP-MM	2.8	3.3	2.8	X	3.3	2.9	0.3
(pg/100ml)	MM-PP	3.0	3.3	3.0	X	3.6	3.2	NS
Cortisol	PP-MM	156.7	140.6	143.2	X	152.4	162.2	12.7
(ng/ml)	MM-PP	167.4	172.4	163.4	X	166.9	157.6	NS
ACTH	PP-MM	15.8	14.2	14.6	X	17.2	17.3	1.8
(pg/ml)	MM-PP	15.5	21.1	20.3	X	13.3	13.2	*

a Statistical significance determined according to multiple Wilcoxon’s test.

* *P*<0.05;

NS, Not Statistically Significant.

Results of physical measurements and clinical blood analysis summarized in Table 3, show that Maca-GO has significant (*P*<0.05) effect on reduction in body weight, lowering both systolic and diastolic blood pressure at simultaneous increase in HDL and serum Iron level.

**Table 3:** Results of measurements and clinical biochemical blood assessment in two groups of perimenopausal women (n=18): One group (PP-MM sequence) receiving for two months daily dose of 2 g (two 500 mg capsules twice daily) of Placebo (PP), followed by crossover (X) after which identical daily dose of Maca-GO (MM) was administered for another two months period (Treatment A - without prior run-in period), and the second group (MM-PP sequence) treated with identical daily doses of Maca-GO for two months followed by crossover and two months of Placebo administration (Treatment B - with prior run-in period)^a^

Hormone	Period on Maca-GO or Placebo (before - and after crossover)	Start of the Trial	without prior run-in period		X	with prior run-in period		SE (±) of Mean (significance) 2
			After 1 month	After 2 month	↑	After 3 month	After 4 month	

Body weight (kg)	PP-MM	69.4	71.1	72.2	X	70.5	69.1	1.41
	MM-PP	69.1	69.1	66.7	X	69.2	69.8	*
Blood Pressure - Systolic (mm Hg)	PP-MM	120.0	123.3	122.2	X	108.8	110.0	3.4
	MM-PP	123.3	118.3	115.0	X	115.0	120.6	*
Blood Pressure - Diastolic (mm HG)	PP-MM	78.9	80.0	68.9	X	67.5	72.9	2.8
	MM-PP	76.7	76.1	74.4	X	80.0	81.3	*
Total Cholesterol (mg/100ml)	PP-MM	209.1	211.4	210.2	X	198.8	209.1	8.64
	MM-PP	217.1	204.4	204.4	X	209.1	217.1	NS
Tri-Glicerides (mg/100ml)	PP-MM	209.1	211.4	210.2	X	198.8	196.1	17.1
	MM-PP	217.1	204.4	204.4	X	209.1	212.0	NS
HDL (mg/100ml)	PP-MM	57.2	62.4	63.2	X	76.3	69.4	3.3
	MM-PP	51.4	59.2	65.0	X	56.0	57.5	**
LDL (mg/100ml)	PP-MM	134.8	139.9	138.9	X	126.3	125.4	9.3
	MM-PP	135.5	135.2	130.8	X	137.7	140.8	NS
Ca (mEq/L)	PP-MM	4.2	4.4	4.5	X	4.6	4.6	0.06
	MM-PP	4.3	4.5	4.8	X	4.6	4.5	NS
P (mg/100ml)	PP-MM	3.0	2.9	3.1	X	3.1	3.1	0.42
	MM-PP	2.6	3.0	2.9	X	2.8	2.7	NS
Fe (mcg/100ml)	PP-MM	58.4	75.1	72.6	X	82.8	89.9	6.17
	MM-PP	58.7	86.0	69.8	X	67.0	79.7	*

a Statistical significance determined according to multiple Wilcoxon’s test.

* *P*<0.05;

** *P*<0.01;

NS, Not Significant.

**Figure 2:**
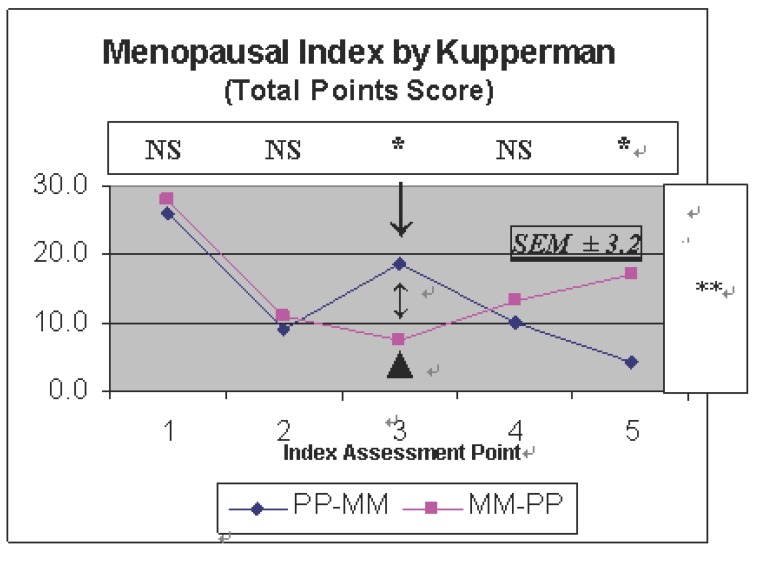
Menopausal Index according to Kupperman as determined in two groups of perimenopausal women (n=18): after administration of 2,000 mg Maca-GO daily at: 1) Start - before the trial; 2) after subsequent one month and 3) two months administration of Placebo or Maca-GO (Treatment A - without prior run-in period), with crossover (marked by arrows) after completion of the Period A, followed by another two months (4 & 5 respectively) of administration of the same quantities of daily dose with the change from Placebo to Maca-GO and vice-versa (Treatment B - with prior run-in period). Symbol “Δ” indicates a crossover (X) point. Statistical significance: The value 3.2 = calculated Standard Error of Mean (SEM ±); Panel above the diagram, indicate difference between Maca-GO and Placebo Treatments (A and B respectively); *P<0.05; **P<0.01 and NS, Not Significant. The right hand side panel, indicates significance of differences between the scores obtained along the subsequent assessment points (between and within the PP-MM and MM-PP Treatments).

In personal interviews conducted by a gynecologist, after already one month into Treatment A, a great majority of women receiving both Maca-GO and Placebo treatment (19 of 20) observed highly significant (*P*<0.01) reduction in feeling discomfort typically observed in the early perimenopausal stage as assessed by answers given to the questions according to Kupperman’s Menopausal Index (KMI) and expressed by the total point score of this Index (Figure 2). While towards the 2nd month of treatment, in Maca-GO group observed further lowering in the KMI score, then, women in Placebo group showed an overall increase in the total point score showing an increase in subjectively assessed menopausal score, indicating an increase in feeling of personal discomfort during the second month on Placebo.

From individually assessed menopausal symptoms (Table 4), after Maca-GO administration, the following were most pronounced at the “before crossover” stage: hot flushes, excessive sweating, and interrupted sleeping pattern - highly significantly improving (*P*<0.01), and to a lesser extend – but statistically-significant (*P*<0.05) improvement was recorded in such symptoms as nervousness, depression and heart palpitations. The pattern of changes in individually-assessed negative menopausal symptoms as mentioned above was reflected in a total menopausal score of all the individual symptoms (as in Figure 2) showing that Maca-GO significantly improved the KMI score, hence, lowering the severity of perimenopausal effect on women.

**Table 4:** Results of Menopausal Index assessment of two groups of perimenopausal women (n=18) according to Kupperman questionnaire: One group (PP-MM sequence) receiving for two months daily dose of 2 g (two 500 mg capsules twice daily) of Placebo (PP), followed by crossover (X) after which identical daily dose of Maca-GO (MM) was administered for another two months period (Treatment A - without prior run-in period), and the second group (MM-PP sequence) treated with identical daily doses of Maca-GO for two months followed by crossover and two months of Placebo administration (Treatment B - with prior run-in period)^a^

Hormone	Period on Maca-GO or Placebo (before - and after crossover)	Start of the Trial	without prior run-in period		X	with prior run-in period		SE (±) of Mean (significance)
			After 1 month	After 2 month	↑	After 3 month	After 4 month	

Hot flushes	PP-MM	9.3	2.2	10.2	X	2.5	0.6	0.58
	MM-PP	7.6	3.1	1.3	X	4.0	8.0	**
Excessive sweating	PP-MM	3.4	1.1	1.3	X	0.8	0.3	0.47
	MM-PP	2.9	0.9	0.5	X	1.5	3.0	**
Interrupted sleep pattern	PP-MM	1.6	0.2	2.4	X	0.8	0.0	0.33
	MM-PP	2.9	0.9	0.5	X	1.0	2.5	**
Nervousness	PP-MM	3.8	2.2	2.0	X	2.5	1.4	0.45
	MM-PP	4.9	2.0	1.8	X	2.3	1.8	*
Depression	PP-MM	1.4	0.7	0.9	X	0.9	0.1	0.19
	MM-PP	1.3	0.4	0.4	X	0.4	0.4	*
Loosing body balance	PP-MM	0.6	0.1	0.1	X	0.1	0.0	0.18
	MM-PP	1.0	0.3	0.3	X	0.5	0.4	NS
General weakness	PP-MM	1.9	1.3	1.2	X	1.4	0.1	0.42
	MM-PP	2.1	1.4	1.0	X	2.0	0.5	NS
Joints pain	PP-MM	0.8	0.2	0.3	X	0.4	0.0	0.31
	MM-PP	1.1	0.2	0.5	X	0.5	0.1	NS
Headaches	PP-MM	1.2	0.6	0.3	X	0.4	0.0	0.54
	MM-PP	1.4	0.4	0.6	X	0.6	0.3	NS
Heart palpitations	PP-MM	1.3	0.2	0.1	X	0.1	0.4	0.23
	MM-PP	1.8	0.8	0.0	X	0.0	0.1	*
Numbness hands & legs	PP-MM	0.7	0.2	0.2	X	0.4	0.0	0.32
	MM-PP	1.0	0.3	0.5	X	0.4	0.0	NS

a Statistical significance determined according to multiple Wilcoxon’s test.

* *P*<0.05;

** *P*<0.01;

NS, Not Significant.

### Period “after crossover”: with prior run-in period

After crossover within the treatments from Placebo to Maca-GO (PP-MM) and *vice versa* (MM-PP), in a group entering Maca-GO treatment, both FSH and E2 significantly increased (*P*<0.05) after one month and leveled for the remaining monthly interval of the study (Figure 1), while women who changed from Maca-GO to Placebo treatment show gradual and steady decrease in FSH concentration (*P*>0.05) with the level above the value at the start of the study. At the end of the second month on Maca-GO, level of FSH was higher than in women receiving Placebo capsules, although the differences were not statistically significant (*P*>0.05).

Change from Placebo to Maca-Go treatment resulted in a significant (*P*<0.01) increase in E2 concentration with significant decrease (*P*<0.05) observed in the group who changed from Maca-Go to Placebo treatment. After two months Maca-GO administration, the level of E2 was significantly higher (*P*<0.01) as compared to both starting point and the Placebo treatment.

Changes in levels of Progesterone followed closely the pattern recorded in E2 and after two months the differences between Maca-GO treatment and Placebo were statistically highly significant (*P*<0.01) as were the differences between Progesterone levels recorded in both groups prior to the crossover and 2 month after administration of either Placebo or Maca-GO.

There were no noticeable (*P*>0.05) changes in LH patterns recorded. After crossover there were no statistical differences (*P*>0.05) observed in changes of concentration patterns as recorded in the thyroid (TSH, T3 and T4) and Cortisol (Table 2). However, the level of ACTH has significantly (*P*<0.05) reduced after change from Maca-GO to Placebo treatment while there was noticeable, but not significant (*P*>0.05) increase in a group changing from Placebo to Maca-GO treatment.

Crossover has not significantly (*P*>0.05) affected body weight of perimenopausal women in relation to pre-crossover measurement, however there was a tendency for Maca-GO reducing and Placebo treatment increasing the body weight of participating women (Table 3). After crossover, Maca-GO had a significant (*P*<0.05) effect on reduction of both systolic and diastolic blood pressure as compared to corresponding readings in Placebo group. Administration of Maca-GO, significantly (*P*<0.01) increased HDL levels, with most distinctive difference observed in the second blood sampling between Maca-GO and Placebo treatment which tend to increase the LDL concentrations. There were no statistically significant changes (*P*>0.05) recorded in Triglycerides, Cholesterol and LDL concentrations which could be attributed to either treatment, although there was a tendency observed in Placebo slightly increasing LDL levels.

Change from Placebo to Maca-GO has visibly, though not significantly (*P*>0.05) increased Iron concentrations. The significant difference (*P*<0.05) was observed between measurements taken at the last blood sampling in PP-MM group and the start of the trial only.

After the crossover from Placebo to Maca-GO treatment the following individually assessed menopausal symptoms were significantly improving (*P*<0.01): hot flushes and interrupted sleeping pattern, and to a lesser extend (*P*<0.05), depression and excessive sweating (Table 4), while change from Maca-GO to Placebo treatment had opposite effect with most pronounced being hot flushes and excessive sweating.

Majority of women (8 of 9), who after Placebo runin period received Maca-GO capsules observed highly significant (*P*<0.05) reduction in feeling of menopausal discomfort as expressed by the total point score of the KMI (Figure 2) with gradual lowering KMI score observed at the last assessment point (two months after crossover). On the other hand women who changed from Maca-GO to Placebo treatment observed gradual, but not significant (>0.05) increase in feeling of menopausal discomfort as expressed by the KMI score, with a severity increasing with the duration of the Placebo treatment.

## DISCUSSIONS

Adopting recommendations by Hodge & Sterner, the LD50 value for Maca-GO determined in this study in standard bioassay on rats, indicates that quantities of up to 7.5 g of Maca-GO per kg body weight have no visible toxic effects on test animals nor resulted in history-pathological changes of critical internal organs which should be classified as abnormal and therefore, it is considered to be safe for oral administration in therapeutic and dietary preparations for humans. The above dose is considerably higher than the 2 g/kg limit determined by the OECD (20) as non toxic and safe for use as dietary supplement. The results obtained in this study confirm previously obtained observations on rats (12) exposed to various levels of Maca-GO (0.75 g/kg and 7.5 g/kg body weight of rats) where no detectable negative physiological, clinical, history-pathological nor toxic effects existed which could be attributed the used doses of Maca-GO and administered to rats during either 28 days or 90 days experimental periods. There were distinctive physiological and clinical differences in responses of female and male rats to the same dose of Maca-GO indicating its gender-specific physiological action.

It has been suggested by Chacon (3) that action of Maca relies on plant sterols, acting as chemicals which trigger chain of biochemical reactions helping the body itself to produce or modulate production of hormones and other compounds, appropriate to the age and gender of person taking it. Results from analytical research conducted so far it appears that Maca does not contain any analytically-determined plant estrogens, or hormones. There is a consensus of opinion that Maca exhibits strong action characteristic to plant adaptogens (6). In this respect, the sterols in Maca may be used by the body with the help of the pituitary to improve adrenal and ovarian (or testicular) functions, and therefore affecting the thyroid, the pancreas, and the pineal gland (which also makes melatonin helping in improvement of sleep pattern as observed in this study). The above may be one of the available explanations why Maca-GO is so much more effective than phyto-estrogens for regulating hormonal balance and making the endocrine glands work better.

In previous papers from this series (11, 12) it has been reported that Maca-GO helps body to balance levels of hormones and alleviates negative physiological and psychological symptoms experienced by women in early postmenopausal stage (11) suggesting its action as a toner of hormonal processes leading to alleviation of discomfort felt by women in early postmenopausal stage and manifested by total score of the Menopausal Index. The present study showed that Maca-GO, when used in perimenopausal women, resulted in number of significant changes as recorded in levels of hormones (FSH, E2, PG and ACTH) and other biochemical indices such as lowering in body weight, both systolic and diastolic blood pressure, and an increase in HDL and Iron levels. This was associated with reduction of the KMI score (Figure 2, Table 4), all of which, may positively affects on overall physiological status of perimenopausal women, hence, improving a health condition and therefore positively influencing their wellbeing status.

Maca exhibits specific, yet not fully understood endocrine effect, ranging from being an energizing plant (3), stimulating reproductive functions (7-9) and balancing hormones (11, 12) as well as alleviating physical, physiological and psychological discomfort associated with menopause in women (1, 5, 6). Since individual active compound(s) which could be biochemically identified as the key active Maca root component(s) responsible for specific therapeutic functionality of Maca root, have not been clearly determined yet, the authors are referring to Maca-GO root in its entirety and cohesive complexity considered as a therapeutic unaltered herb with its historically-acknowledged and “traditionally-unquestioned” dietary and medicinal properties (1-3). It is reasonable to suppose that the complexity and uniqueness of components present in Maca root such as sterols (campesterol, stigmasterol and beta-sitosterol), polyunsaturated acids and their amides, called “macaenes” and “macamides” (3, 9), aromatic glucosinolates (14) and several alkaloids and others constituents of Maca – yet to be characterized, through their complex synergistic and/or interactive action, will eventually one day provide an answer to specific physiological action at specific doses of standardized Maca preparations recommended for prophylactic and/or specific therapeutic effect for men and women.

According to Dini (14), reported in a literature aphrodisiac powers of Maca for men and women, may be ascribed to presence of prostaglandins and sterols in the hypocotyls of Maca and overall fertility enhancing properties may be attributed to the presence of biologically-active aromatic isothiocyanates derived by hydrolysis of the glucosinolates and specifically due to benzyl isothiocyanate and p-methoxybenzyl isothiocyanates (15). In addition, benzyl isothiocyanate present in Maca root has been reported to be a potent cancer inhibitor of mammary gland and stomach (16).

Observed in this study, effect of Maca-GO on an increase in both FSH and estradiol level which was accompanied by an increase in progesterone level may be compounded by pre-arranged in advance timing of visits for blood sampling, which was not done in precisely identical point of the menstrual cycle. In this study, due to the fact that monthly interviews were conducted by a doctor on groups of women according to schedule of monthly appointments, it was not possible to synchronize the time of blood sampling in precisely the same stage of a menstrual cycle of the each individual women. This factor which may substantially influence concentration of hormones determined in various points of cycle, needs to be rectified in further study planned to be conducted in the future on perimenopausal women. An elevation in progesterone concentration could be a factor responsible for keeping fluctuations in estradiol level at uniformly moderate level in group receiving Maca-GO capsules in both sequences of application: without and with prior run-in periods. The observed relationship between progesterone and estradiol is in accord with observations based on clinical experience, by Lucille (21) who emphasized that the balance between progesterone, estradiol and thyroid function is one of the key factors in female maintaining her hormonal balance during the reproduction years and in menopause. It is a key function of progesterone to control estradiol and prevent negative effects of its dominance as well as to support thyroid function in maintaining growth, healthy bone metabolism and balancing psychological equilibrium in females during and after their reproductive stage. In this study such a relationship has not been confirmed, since Maca-GO administration resulted in both estradiol and progesterone elevation with no significant effect on thyroid profiles in perimenopausal women enrolled in the study.

Reviewing published technical reports, it appeared that Maca preparations tend to be more effective for menopausal patients as compared to treatment with other phyto-estrogenic herbs (4). This was particularly so, when Maca was used in conjunction with nutritional supplements, to wean women (some of them on HRT for many years) off of hormone replacement therapy. For those women who still had some symptoms, a combination protocol involving Maca extract and a minute amount of natural estrogen together with natural progesterone is suggested, which, unlike progestin, is considered not carcinogenic. In the USA health care practitioners increasingly integrate herbal therapies into their medical practice and amongst of phyto-preparations they recommend, Maca by now, is becoming an available alternative to prescription drugs for effective relief of menopausal symptoms (4). As reported by Muller (5), depending on circumstances which are almost always dependent on the dosage involved, Maca may exhibit a “stimulating” or a “balancing” effect on organism. Hence it is recommended to start with a small dose of guaranteed potent and non-toxic whole processed root Maca product and increasing its intake gradually, as needed. The advice to menopausal women is to work with practitioners who can order tests to establish base line hormone levels before starting the Maca therapy, with a follow up with a second series of hormone tests some two months later. This in order to find out if the dose the patient is taking is sufficient and most appropriate to the particular physiological status of women.

For women with menopausal symptoms, most of them will need a minimum of three to four 500 mg capsules daily. However, the very sensitive menopausal woman may only need 2 capsules daily. Dosage can be increased on a weekly basis if this amount is not sufficient until the optimum (minimum effective) dosage is found. Taken at the correct dosage, which differs for each woman, Maca may be able to reduce or completely eliminate hot flashes in as little time as 4 days to a week (5). It is important to mention that, if the dosage is too high - and some women are very sensitive-the Maca will have a stimulating and not balancing effect, and will actually increase the amount of hot flashes. If this happens, Maca users can cut the dosage in half for a week, and then re-evaluate. If the problem persists, cutting the dosage in half again may help to identify the optimal dosage (5).

Other measures women can take to avoid estrogen dominance include eating fiber-rich diets (excess estrogen will be carried out with the bowel movements), exercising (aerobic exercise lowers estrogen level), and having sufficient Omega 3 fats (including ground flax seeds or oil and fatty fish in their diet).

The estradiol levels observed in this study were above 30 pg/ml – typical to levels indicating pre- or perimenopausal stage with functional or slightly affected ovaries - a status which is observed in women at the time of entering a peri-menopausal stage of life. According to Malespina (10), levels of 30 pg/ml and above (with average 60-75 pg/ml levels) are considered as adequate for woman to avoid symptoms of discomfort characteristic to menopause. On the basis of results reported in this paper and literature available so far, it is reasonable to assume that Maca-GO, although by itself containing no plant hormones, through regulating the organs of internal secretion, such as the pituitary, the adrenal glands, the pancreas, etc. stimulated and/or contributed to regulatory mechanism responsible for optimizing ovarian functions and secretion of the quantity of estrogen – well above the required minimum – as observed in this study. This may contribute to reduction in feeling of menopausal discomfort by women taking Maca-GO and avoiding problems linked to mental, physiological and physical symptoms responsible for discomfort associated with perimenopause and manifested by results in individual symptoms measured in KMI (Table 4) and expressed by overall KMI score (Figure 2).

Remembering that Maca-Go does not itself contain any hormones (1-5), the action of Maca root in its coherent unique complexity of integral active constituents such as alkaloids, sterols, glucosinolates, amino acids, fatty acids and minerals (13-16), and not selectively extracted groups of components such as macamides and macaenes (9) with elimination of remaining constituents – considered as complementary- or synergistically-essential, act on the body by stimulating pituitary into producing and secreting the precursor hormones which in tern rise oestrogen, progesterone and testosterone levels, with a simultaneous help in balancing the adrenal glands, the thyroid and the pancreas. Therefore, it is reasonable to suppose that Maca appears to regulate ovarian function rather than stimulating them as observed in other phytoestrogenic preparations based on black cohosh, soy, red clover and other.

Research evidence available from some North American and English scientists who were studying the pathologies of people living at high altitudes of Andes (at about 4,000 meters), characteristic to the altitude, where those people cultivating Maca live, show that women residing there, had no postmenopausal problems (5). Various commercial Maca preparations are now available on the market in the USA and Europe for those practitioners, who, based on their personal clinical experience and limited research conducted so far, may recommend Maca for use by women for reasons such as hormone balance, for energy, for PMS, for menopausal symptoms, including hot flashes, vaginal dryness, the “blues”, for thyroid health, immune balance, and nutritional support for postmenopausal women to maintain healthy bones (4, 5, 10).

Obtained results and available literature evidence may suggests that Maca-GO, when taken in a right dose is acting as a toner of hormonal processes which may alleviate discomfort felt by women during perimenopausal stage.

Observations made in the pilot clinical study reported here justify further more complex study on use of Maca in perimenopausal women. This in order to assess effectiveness of Maca as a non-hormonal therapeutic supplement which may help women to reduce discomfort associated with menopause as an alternative to, or helping to reduce dependence on HRT programs.

In previous study on laboratory animals (12) it has been observed that in both, high and low levels of Maca-GO significantly lowered cortisol level, while ACTH level was, distinctively, although not significantly increased at low dose and distinctively lowered at the high Maca-GO dose. This observation indicated that anti-depressive effect was a dose-related response during short-term administration. However, during an extended use of Maca-GO (90 days), both cortisol and ACTH were substantially lowered at both administration levels, which could be an indication of its positive effect on reducing symptoms of depression frequently affecting women in their perimenopausal stage.

On the basis of results obtained in laboratory model study and on human subjects, De Moranvile & Jacson (22), Sapolsky (23) demonstrated close association between an increase in both cortisol and ACTH levels and the state of depression. Results obtained in the present study have not confirmed the above relationship since Maca-GO had no effect on cortisol level but significantly increased serum ACTH concentrations (Table 2) with overall decrease in severity of depression observed as a result of both Maca-GO and Placebo treatment (Table 4). This indicates that women reacted visibly responding to Placebo treatment in similar manners as to Maca-GO treatment – which was most distinctively evident in FSH and E2 and number of individual menopausal symptoms such as reduction in frequency and severity of hot flushes, excessive sweating, interrupted sleep pattern, nervousness depression and headaches (Table 4) and expressed by the overall Kupperman’s Menopausal Index score (Figure 2). However, when placebo effect was eliminated by switching treatments at a crossover point, at which results were recorded with prior run-in period, then, there was nearly straight-linear reduction in severity of menopausal symptoms induced by Maca-GO treatment (Figure 2), while Placebo resulted in a simultaneous increase in severity of the same symptoms as reflected by the KMI score, with a significant difference existing between treatment at the end of the 2 months “after crossover” treatment. This may suggests that Maca-GO as a dietary supplement may help women in alleviating depression and related psychological and physical symptoms of discomfort associated with, and frequently experienced by women in perimenopausal stage. Studies by other authors (3) also indicate that Maca can be helpful in reducing discomfort caused by menopausal symptoms and limited case studies on laboratory animals have shown that Maca can be effective for premenstrual syndrome (PMS) as well (1).

Results obtained in this study have not confirmed the previously observed in laboratory trials (12) where Maca-GO had positive effect of on reduction in blood cortisol indicating possible positive effect of Maca-GO on lowering susceptibility of rats to stress factors and its sedative effect on laboratory animals, the properties also reported by Lopez Fondo *et al*. (24). Also, observed in laboratory study the effect of Maca-GO administration on maintaining, or slight reduction of Estradiol level, at simultaneous increase in Progesterone has not been observed in the present work. There was an expectation that application of Maca-GO during perimenopausal phase in women, may counteract reported (25) tendency in gradual increase in Estradiol level due to decline in secretion of Progesterone. According to Stahl (26) a gradual increase in blood Estradiol level observed in perimenopausal stage leads to development of depression but in this study with Maca-GO administration, resulting in elevation of both estradiol and progesterone, women participating in the study, amongst other positive responses, reported a reduction in severity of depression. It appears that application of Maca-GO may slow down, delay or prevent depression and other unpleasant symptoms which are manifested prior to and/or during the menopause (4, 5, 11) at elevated both estradiol and progesterone levels. In a preliminary study on ovariectomized rats (27) it has been suggested that Maca-GO possesses anti-depressant-like and sedative, but not anxiolytic effects as measured in locomotor activity test, Porsolt and anxiolytic activity tests which corresponded with significant lowering in Cortisol and ACTH concentrations, leading to conclusion that Maca-GO could have value in treatment of some depressive symptoms during perimenopausal period. The follow-up study (28) on the same samples of Maca-GO as used in this study, when tested against Fluoxentine, a known antidepressant agent, confirmed the above assumptions showing that Maca-GO possesses typical antidepressant–like characteristics. After Maca-GO administration to ovariectomized rats, both blood Cortisol and ACTH as well as spontaneous activity and immobility time (Porsolt test) were significantly (*P*<0.05) reduced, while Fluoxetine induced anti-depressive effect in control, non-ovariectomized animals only, without affecting ovariectomized rats, with one exception, that, Fluoxetine increased the blood Cortisol only in non-ovariectomized rats without significantly affecting ACTH and spontaneous activity test values. This led to conclusion that antidepressive action of Maca-GO is based on different mode of action in non- and ovariectomized rats as compared to the antidepressive effect of Fluoxentine which can be translated to expected different responses of pre- and post-menopausal women to Maca-GO. The above results from a model laboratory tests (27, 28) in conjunction with the results recorded in this study on perimenopause women, suggest that active phyto-components present in Maca-GO act in a specific way on release of body steroids or affecting the hypothalamo-pituitary-ovarian axis in women resulting in triggering similar, but other than serotoninergic response mechanisms as induced by Fluoxentine resulting in its anti-depressive action demonstrated on rats (28). Further study would be needed to clarify those mechanisms.

In previous model laboratory study with the use of Maca-GO in rats (12) an increase in level of blood glucose was observed which could explain reported energizing effect of Maca and its use as an energizing dietary supplement for sport people and those whose lifestyle requires energy reserves for intensive physical activity (4, 26, 29, 30). The results from this study have not confirmed Maca-GO having positive energizing effect on perimenopausal women since there was no significant reduction in the individual score of the “General Weakness” symptom as listed in Kupperman’s Menopausal Index, which indicates that there was no energizing effect of Maca-Go on perimenopausal women participating in this study. Observed in this study positive effect of Maca-GO on serum Iron level is in accord with results obtained in a laboratory model trials on rats (12) and reported in a literature (31), indicating that Maca may play an important role in stimulation of the absorption of dietary Iron from the digestive tract. Maca-GO has not visibly affected level of serum Calcium and Phosphorus in this study.

Results reported from this study support observations from previous research on Maca-GO (11, 12) and earlier study of Muller (5) that Maca, depending on the level and the length of its intake, may act either as an adaptogenic herb displaying “stimulating” or “balancing” effect. Therefore, in the further study, it will be essential to establish ranges of quantitative administration of Maca-Go, which will act for certain as a stimulating or balancing dietary supplement for well-defined groups of subjects in their specific physiological state (gender, age, weight etc.) for specific purpose and expected functionality.

## CONCLUSIONS

1. In comparison to placebo, After 2 months of using Maca-GO capsules by perimenopausal women, serum levels of FSH, E2, PG and ACTH substantially increased.
2. Results of interviews conducted by a gynecologist after two months of using Maca-G supplement, majority of women (15 of 18 which concluded the four month trial) observed reduction in general feeling of discomfort, typically observed in the early postmenopausal stage.
3. According to responses given in Kupperman’s questionnaire for assessment of Menopausal Index, frequency of hot flushes and incidence in night sweating, interrupted sleep pattern, nervousness, depression and heart palpitations, were most pronounced symptoms in improving quality of life of perimenopausal women exposed to Maca-GO administration.
4. Preliminary observations outlined in this paper justify further clinical study on use of Maca-GO in perimenopausal women, so as to assess effectiveness of Maca as a potential non-hormonal therapeutic supplement which may help women to reduce discomfort associated with perimenopause as an alternative to, or lessening dependence on HRT program.
